# Increased Granulopoiesis in the Bone Marrow following Epstein-Barr Virus Infection

**DOI:** 10.1038/s41598-019-49937-w

**Published:** 2019-09-17

**Authors:** Yasuhiro Katahira, Hiroshi Higuchi, Hiromichi Matsushita, Takashi Yahata, Yuichiro Yamamoto, Ryo Koike, Kiyoshi Ando, Katsuaki Sato, Ken-Ichi Imadome, Ai Kotani

**Affiliations:** 1 Department of Hematological Malignancy, Institute of Medical Science, Tokai University, 143 Shimokasuya, Isehara, Kanagawa 259-1193 Japan; 2 Research Institute of Science and Technology, Tokai University, 4-1-1 Kitakinme, Hiratsuka, Kanagawa 259-1292 Japan; 3 Division of Pathology and Clinical Labolatories, National Cancer Center Hospital, Tokyo, 104-0045 Japan; 4 Division of Hematopoiesis, Research Center for Regenerative Medicine, Tokai University School of Medicine, Isehara, Kanagawa 259-1193 Japan; 5 Department of Oral and Maxillofacial Surgery, Nihon University School of Dentistry, Tokyo, 101-8310 Japan; 6 Department of Hematology/Oncology, School of Medicine, Tokai University, 143 Shimokasuya, Isehara, Kanagawa 259-1193 Japan; 7 Division of Immunology, Department of Infectious Diseases, Faculty of Medicine, University of Miyazaki, Miyazaki, 889-1692 Japan; 8 Japan Agency for Medical Research and Development (AMED), Tokyo, 100-0004 Japan; 9 Department of Advanced Medicine for Infections, National Center for Child Health and Development (NCCHD), Tokyo, 157-8535 Japan

**Keywords:** Infection, Viral infection

## Abstract

Epstein-Barr virus (EBV) is associated with several disorders. EBV is known to modulate the proliferation and survival of hematopoietic cells such as B cells and T cells in human. However, the effects of EBV on hematopoiesis itself have not been investigated. To study EBV infection in murine models, their hematopoiesis must be humanized, since EBV infection is limited only in primates. To engraft the human hematopoiesis, NOD/Shi-*scid*-IL2rγ^*null*^ (NOG) mice were used. Usually, the hematopoiesis humanized mice reconstitute only lymphoid cells, but myeloid cells are not. However, we revealed human macrophages (hMφ) and their precursor monocytes were increased in peripheral tissues of EBV-infected mice. Furthermore, our previous report indicated Mφ accumulation in spleen was essential for development of EBV-positive tumors, suggesting that EBV modulates human hematopoiesis in order to thrive. Interestingly, we revealed a dramatic increase of immature granulocytes only in bone marrow of EBV-infected mice. In addition, GM-CSF, a cytokine that is essential for differentiation of the myeloid lineage, was significantly increased in EBV-infected mice. These results were also reproduced in patients with EBV-related disorders. We suggest that the hematopoietic alterations during EBV-infection might contribute immune suppression to the development and exacerbation of EBV-related disorders.

## Introduction

An immune system is essential for organisms to overcome invasion by pathogens such as viruses and microbes and is also required for safeguarding against toxic substances. The vertebrate immune system has developed both innate and adaptive responses, which together provide their hosts with strong protection from foreign insults^1–4^. However, many viruses use immune suppression in order to persist and replicate in their host. For example, herpes simplex virus 1 (HSV-1) abrogates the antiviral activity of the host protein, hZAP^5^ and human cytomegalovirus (HCMV) suppresses the antiviral immune response by inducing IL-10 expression in infected human CD4^+^ T cells^6^. In addition, alphaviruses utilize the 3′ non-translated region (NTR) of their genome to evade the immune system by inhibiting replication of macrophages (Mφ) and dendritic cells (Dc)^7^.

Epstein-Barr virus (EBV), is a herpes virus that infects selected primates, including humans^8,9^. EBV escapes immune surveillance by miRNA-driven inhibition of the CD4^+^ T-cell antiviral response^10^. EBV was discovered and isolated from Burkitt’s lymphoma and is recognized as the cause of infectious mononucleosis, B-cell lymphoma, NK/T-cell lymphoma, nasopharyngeal carcinoma (NPC), and several other disorders^10,11^. Globally, more than 90% of adults are infected with EBV^11,12^. Although EBV infection is relatively benign under normal conditions, it can cause cancer in some individuals and this has been related to its immune-suppressive activities^13,14^. In some B-cell lymphomas, EBV-positive cases have a poorer prognosis than those that are EBV-negative^15,16^. Furthermore, NPC and NK/T-cell lymphoma are frequently observed in East Asia^17^ also have a poor prognosis. Accordingly, extensive study of the molecular mechanisms by which EBV exerts pathogenic effects is a high priority. A murine model of EBV infection has been established by humanizing the hematopoietic system in mouse^18^. Using this model, we showed that exosomes, which are secreted vesicles, are derived from EBV-transformed lymphoblastoid cell lines (LCLs) and contain EBV-encoded miRNAs; these miRNAs induced an immune regulatory phenotype in Mφ and exacerbated tumorigenesis^19^. Human myeloid cells are not well developed in hematopoietic humanized mice because several mouse murine cytokines, such as IL-3 and GM-CSF, do not act on the human cognate receptors^20,21^. On the other hand, infection of these mice with EBV significantly increases the number of infiltrating human macrophages in lymphoma tissue, which suggests that EBV infection could subvert host hematopoiesis to facilitate its persistence.

In this study, we evaluated the influence of EBV infection on hematopoiesis, especially focusing on myeloid cell differentiation. Tissue resident macrophages have different origins and potential for self-renewal in specific tissues^22^. For example, microglia in the brain, and macrophages in the liver, lung, and epidermis are thought to differentiate from progenitors in the yolk sac and fetal liver during embryonic development, respectively. In contrast, macrophages in the gut, dermis, heart, and pancreas are derived from blood monocytes that differentiate in the bone marrow (BM). In the humanized mouse model, hematopoietic stem cells (HSCs) in the BM are suggested to be the sole source of mature human hematopoietic cells. By extension, this indicates that BM monocytes are the main source of human tissue Mφ. Consistent with the increased number of Mφ in lymphoma tissue, the ratio of peripheral blood monocytes (hCD45^+^, hCD14^+^) is increased in EBV-infected mice over in non-infected mice. Furthermore, the granulocytic cell population is dramatically increased in the BM of EBV-infected mice. Interestingly, the increase in granulocytes is accompanied by higher number of immature progenitors in the BM of EBV-infected mice compared with non-infected mice. This increase in immune progenitors correlated with elevated levels of human GM-CSF, cytokine that support myeloid development. These results are mirrored by the status of human patients with EBV-related disorders.

Immature myeloid cells with immune suppressive function are also referred to as ‘myeloid-derived suppressor cells’ (MDSCs) and are reported to support survival of tumor cells in some tumor models. Although we did not observe such an immune suppressive function of EBV-infected immature granulocytes, it was shown that EBV had a potential to induce aberrant granulopoiesis in this study. Taken together, we introduce EBV-induced hematopoietic alteration in BM and suggest that the process may be a cause of virus-mediated immune suppression; this would exacerbate the pathology of EBV-related disorders.

## Result

### EBV infection increases the number of human peripheral blood monocytes

Since EBV is infected with only primates, we established humanized NOG mice which were transplanted by human CD34^+^ cord blood. After the expression of human CD21 which is the essential molecules for EBV infection on human CD19 positive B cells was observed in three months, 1 × 10^3^ of 50% transforming dose of EBV was injected from the tail vein. It takes 6 weeks to develop the lymphoproliferative disease in those model.

**Figure 1:**
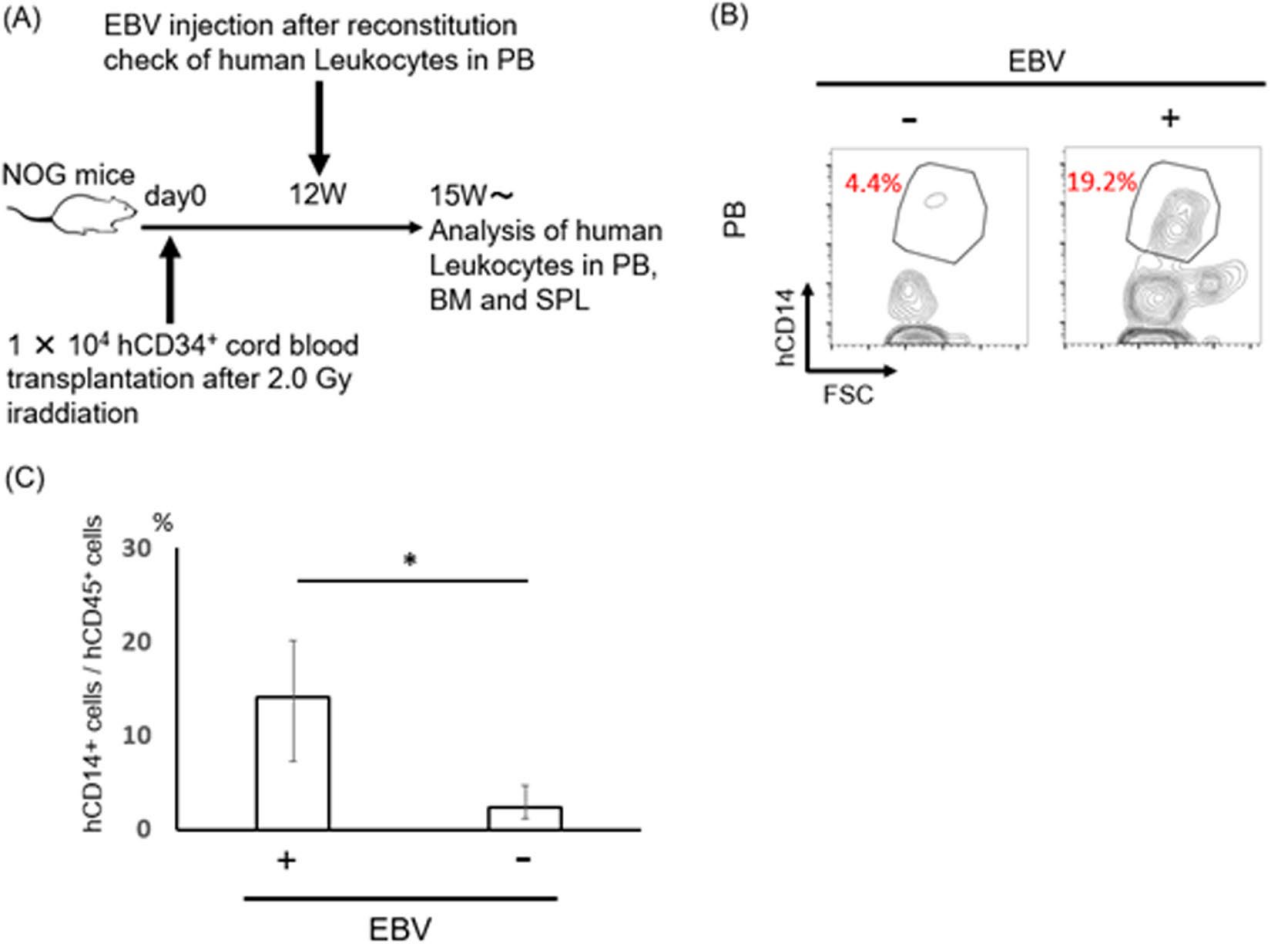
Peripheral alteration of immune cells seemed to be occurred from bone marrow in EBV infected mice. (**A**)Schematic view of a process of the analysis. For the reconstitution of human hematopoiesis in NOG mice, 1 × 10^4^ human CD34^+^ cord blood cells were transplanted into NOG mice after irradiation (2.0 Gy). EBV injection was performed after confirming the engraftment of human leukocytes in mice. Hematopoietic analysis was performed after beginning dramatically weight loss. Uninfected mice were utilized as controls. (**B**,**C**) Comparison of human Monocyte between infected (n = 4) and uninfected (n = 7) humanized mice in PB. hCD14^+^ monocytes significantly increased in EBV infected mice. hCD14^+^ were detected from hCD45^+^ population. **p < 0.05.

Using the murine EBV^+^ LPD model^19^, we observed that EBV infection dramatically increased the infiltration of hMφ (CD163 positive cells and CD68 positive cells) in the spleen which were proved to support the survival of EBV infected cells (EBER positive cells). Since hMφ are derived from blood circulating monocytes in the humanized mouse model, we compared the ratio of monocytes (CD14 positive cells) in peripheral blood (PB) between EBV-infected and non-infected mice. There was significant increase in the number of monocytes in EBV-infected compared with that in non-infected mice (Fig. 1B,C). In contrast, other lineage cells such as T cells (CD3^+^ cells) and B cells (CD19^+^ cells) did not show statistically significant increase in the PB in EBV-infected mice compared with that in EBV-non-infected mice. These results suggested that EBV infection modulates granulopoiesis in this humanized mouse model.

### EBV infection affects bone marrow hematopoiesis

**Figure 2:**
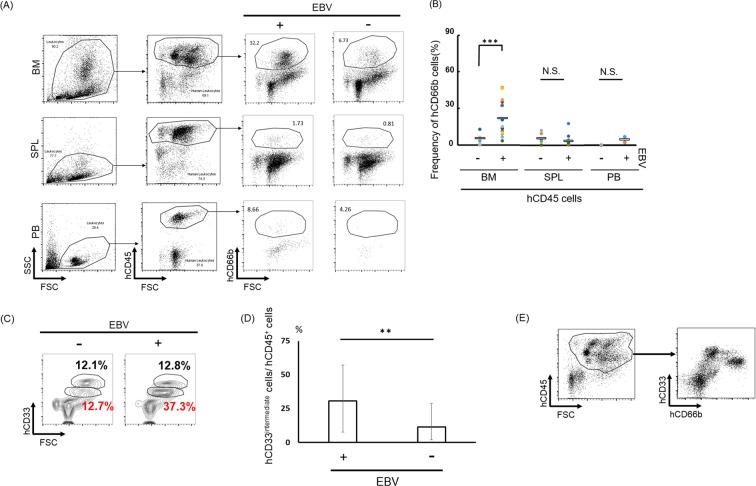
Development of granulopoiesis only in bone marrow with EBV infection. (**A**) hCD45^+^ and hCD66b^+^ Abs were used for detection of granulocyte by flow cytometer. Bone marrow (BM) (EBV^+^; n = 16, EBV^−^; n = 6), SPL (spleen) (EBV^+^; n = 12, EBV^−^; n = 6) and PB (peripheral blood) (EBV^+^; n = 5, EBV^−^; n = 2) in mice were analyzed. (**B**) Frequency of granulocyte in humanized mice tissue. BM, SPL, PB were compared between infected and uninfected condition. Data are mean ± SD. (**C**,**D**) hCD33^intermediate^ cells n BM between EBV-infected (n = 10) and uninfected (n = 5) humanized mice BM. hCD33^intermediate^ cells were detected from hCD45^+^ cell population. **p < 0.05. (**E**) The majority of CD33^intermediate^ cells are hCD66^+^.

To investigate the effects of EBV infection on hematopoiesis, we compared cellular BM components of EBV-infected and non-infected mice using flow cytometry (Fig. S1). This revealed that the human CD66b (hCD66b)^+^ cells were dramatically increased in EBV-infected murine BM before the onset of disesases (Fig. 2A,B). The hCD33^intermediate^ granulocytic population was also significantly increased in the BM of infected mice (Fig. 2C,D). The majority of hCD33^intermediate^ cells population was CD66b positive (Fig. 2E).

These results suggest that granulopoiesis was dramatically stimulated in the BM by EBV infection.

### Early but not late granulopoiesis is induced by EBV infection in the bone marrow

**Figure 3:**
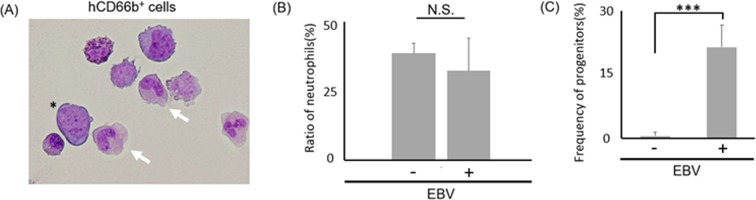
Early but not late granulopoiesis is induced by EBV infection in the bone marrow. (**A**) May-Grunwald Giemsa stain of hCD66b^+^ cells which were isolated from mice BM. Blast (*) and matured cells were observed (x600). White arrows show band neutrophils. (**B**) Ratio of band/segmented neutrophils in isolated hCD66b^+^ cells of BM were compared between infected (n = 3) and uninfected (n = 3) mice. Significant difference were not detected (Not significant; N.S.). (**C**) Frequency of hCD66b^+^ myeloid progenitors in BM were compared with infected and uninfected mice. EBV infected mice BM showed significantly increased progenitors. Data mean ± SD. ***p < 0.005.

While, in the BM, robust increase of hCD66b^+^hCD33^intermediate^ cells were observed, they were barely detected in the periphery of EBV-infected mice, suggesting the possibility that differentiation of granulocytes were dysregulated at some stage. To investigate the differentiation of the increased hCD66b^+^ population, we observed the morphology of hCD66b^+^ cells isolated from infected murine BM (Fig. 3A). The abundance of mature neutrophils (such as band neutrophils and segmented neutrophils) was not significantly different between infected and non-infected mice (Fig. 3B, Table S1), while the number of immature granulocytes was increased in EBV-infected mice BM (Fig. 3C), suggesting that early but not late granulopoiesis is induced by EBV infection. In several cancers, malignancies are exacerbated by the presence of myeloid-derived suppressor cells (MDSCs), which are heterogeneous immature BM cells^23–25^. To investigate whether the detected hCD66b^+^ population contained MDSCs, we analyzed the expression of hCD33 and hHLA-DR by flow cytometry^25^ and detected an hCD33^+^hHLA-DR^−^ MDSC-like subpopulation (Fig. S2A). Then we performed a human T-cell proliferation assay to confirm whether the MDSC-like population has immuno-suppressive activity. T-cells were stimulated by anti CD3/ CD28 dynabeads. Then coculture of the several ratios (1:0, 8:1, 2:1, 1:1) of human T cells to murine BM cells were analyzed. As a result, proliferation of T-cells (Both CD4^+^ and CD8^+^ population) was not suppressed by co-culture with the MDSC-like cells in any ratio of the mixture (Fig. S2B). These results suggest that the MDSC-like population does not have immuno-suppressive activity.

### Production of hGM-CSF is enhanced by EBV infection

**Figure 4:**
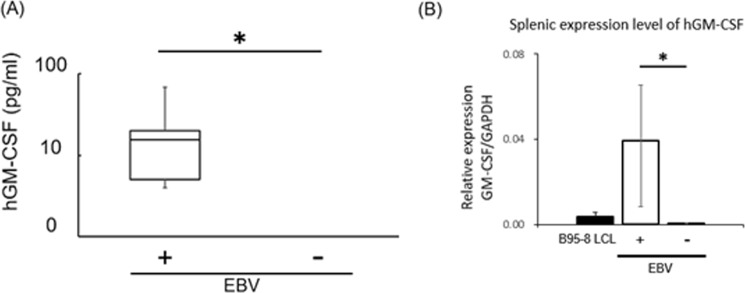
hGM-CSF was increased in EBV infected mice. (**A**) hGM-CSF in mice plasma were quantified by ELISA assay. Experimented number of infected mice = 7, uninfected mice = 3. Data are mean ± SD. Lower limit of detection (LLD) is 0.14 pg/ml. 2/3 of uninfected mice values are indicated as “ND” due to the values less than LLD. **p < 0.05. (**B**) Splenic expression of hGM-CSF in EBV infected mice (N = 3) and uninfected mice (N = 3) was analyzed by q-PCR. hGAPDH was used as internal control for quantification of hGM-CSF. **p < 0.05.

Human GM-CSF (hGM-CSF), but not its murine equivalent, is required for the differentiation of human granulocytes. To analyze the level of secreted hGM-CSF, we performed an ELISA using plasma collected from infected and non-infected mice (Fig. 4A, Table 1). A significant amount of hGM-CSF was detected in the plasma of EBV-infected mice before the onset of disease, but not in non-infected mice.

**Table 1:** Serum levels of GM-CSF in hematopoietic humanized mice with EBV infection or uninfected. Human GM-CSF antibody was used to evaluate GM-CSF amount in mouse model by ELISA assay. The data are expressed as the mean value in each mouse.

Sample No.	EBV Injection	LPD	hGM-CSF^※^(pg/ml)
1	+	+	15.30
2	+	+	18.40
3	+	+	6.33
4	+	+	3.61
5	+	+	1.96
6	+	**−**	21.10
7	+	**−**	67.50
8	**−**	**−**	ND
9	**−**	**−**	0.03
10	**−**	**−**	ND

^※^As determined by hGM-CSF specific ELISA.

Next, to investigate the production of of hGM-CSF in SPL, we performed q-PCR for hGM-CSF, because the previous reports demonstrated EBV-infected B-cells produce hGM-CSF^19^. As was expected, the mRNA for hGM-CSF prominently increased in the SPL of infected compared with that in non-infected mice (Fig. 4B).

### Upregulation of GM-CSF and increased granulopoiesis in human patients with EBV-related disorders

**Figure 5:**
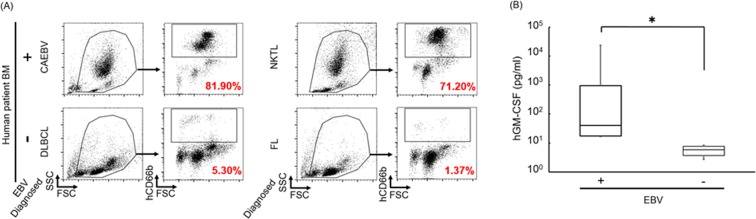
Increased GM-CSF and granulopoiesis were detected in hematopoietic disorder patients with EBV infection. (**A**) Comparison of granulocyte population in BM between EBV-infected and uninfected patients. Anti-human CD66b Ab was used for the detection. Collected EBV^+^ patient’s samples were CAEBV (n = 1), NKTL (n = 2), and EBV^-^ patient’s samples were DLBCL (n = 1), FL (n = 2) patients, respectively. Formal name of each abbreviations is bellow; CAEBV = Chronic active EBV infection, NKTL = NK/T-cell lymphoma, DLBCL = Diffuse large B-cell lymphoma, FL = Follicular lymphoma. Representative data are indicated. (**B**) ELISA assay of hGM-CSF in serum of patients. EBV^+^ data were detected from HLH (N = 8) and PTLD (N = 4) patients, EBV^−^ data were detected from sJIA (N = 2) and KD (N = 4) patients. Data are mean ± SD. Significant difference was detected between EBV infected patients and uninfected patients. HLH = Hemophagocytic lymphohistiocytosis, PTLD = post-transplantation lymphoproliferative disorder, sJIA = systemic juvenile idiopathic arthritis, KD = Kawasaki disease. *p < 0.05.

As EBV infection caused increased granulopoiesis in the mouse model, we next determined whether this was recapitulated in humans by comparing BM hematopoiesis and GM-CSF secretion in EBV-associated LPD patients with that of control subjects. We found that the BM in EBV-infected patients such as chronic active EBV infection (CAEBV) and extranodal NK/T-cell Lymphoma (ENKL) patients contained an increased CD66b^+^ population (Fig. 5A). In contrast, the hCD66b^+^ population could not be detected in the BM of the control group, which comprised patients with EBV-negative malignancies such as follicular lymphoma (FL) and diffuse large B-cell lymphoma (DLBCL). Furthermore, GM-CSF was also significantly increased in the PB of patients with EBV-related disorders (Fig. 5B, Table 2) compared with controls. These results suggest that the EBV-dependent altered granulopoiesis mouse model recapitulates key elements of the pathology found in patients with EBV-related disorders.

**Table 2:** Serum levels of GM-CSF in human patients with EBV infection or uninfected. Human GM-CSF antibody was used to evaluate its amount in human patients by ELISA assay. The data are expressed as the mean value in each patient.

Patient No.	Infection	Disorder	hGM-CSF^※^(pg/ml)
P1	+	HLH	57.2
P2	+	HLH	11.0
P3	+	HLH	22.6
P4	+	HLH	18.9
P5	+	HLH	13.0
P6	+	HLH	12.4
P7	+	HLH	360.0
P8	+	PTLD	23.0
P9	+	PTLD	23992.0
P10	+	PTLD	2707.0
P11	+	PTLD	4895.0
P12	+	PTLD	96.8
C1	**−**	sJIA	7.1
C2	**−**	sJIA	7.8
C3	**−**	KD	3.4
C4	**−**	KD	8.5
C5	**−**	KD	4.6
C6	**−**	KD	2.4

^※^As determined by hGM-CSF specific ELISA.

## Discussion

Many studies indicate that viral infection alters host immunity^5–7^. We and others demonstrated that immune regulatory Mφ exacerbate the malignant phenotype^19,26,27^. In a previous study, we reported that human Mφs, which were poorly developed in non-infected mice, could support EBV^+^ B-cell lymphoma in an exosome-dependent manner. Here, we extend these findings by showing that human monocytes are also increased in the PB of EBV-infected mice compared with non-infected mice. Our data suggest that EBV induces a comprehensive rewiring of human hematopoiesis. In support of this, we found that hCD66b-positive cells were dramatically increased only in the BM but not in PB and SPL. hCD66b is expressed in myelocytes, metamyelocytes, and mature neutrophils such as band and segmented neutrophils. Mature neutrophils were barely found both in EBV-infected and non-infected mice, while immature granulocytic progenitors were significantly increased in the BM of EBV-infected mice. In those immature progenitors, cell types including myeloblasts, monoblasts and myelocytes were observed (Fig. S3, Table S1) suggesting that EBV supports early but not late maturation of granulocyte.

Differentiation of myeloid cells is promoted by cytokines such as G-CSF, GM-CSF, and IL-3. While G-CSF has cross-activity in both mice and humans, GM-CSF and IL-3 do not. Transduction of human GM-CSF and human IL-3 enhances reconstitution of the human myeloid cell line in hematopoietic humanized mice^28^. Therefore, it is expected that human GM-CSF and IL-3 are secreted in infected mice. Although human IL-3 expression was not detected in whole BM cells of infected mice (data not shown), the ELISA showed significantly increased secretion of human GM-CSF in the plasma of EBV-infected mice compared with in that of EBV-uninfected mice.

human GM-CSF was upregulated in the spleen of EBV-infected mice. This result is consistent with a previous study that reported GM-CSF secretion by EBV-infected B-cells^29^. It is generally recognized that humanization in NOG and NSG mouse compromises myelopoiesis due to the insufficient expression of several human cytokines and growth factors such as M-CSF, IL-3, thrombopoietin and GM-CSF. EBV support only early granulopoiesis but not late granulopoiesis. Thus, enhanced expression of human GM-CSF might partly explain the increase of CD66b^+^ cells in the BM of EBV-infected mice.

Tumor initiation and maintenance can be promoted by myeloid-derived suppressor cells (MDSCs)^30^, which are thought to be immature myeloid progenitors that are produced by abnormal differentiation. We detected an MDSC-like population (hCD45^+^, hCD33^+^, hHLA-DR^−^) in BM and SPL of EBV-infected mice. Previously, it was reported that MDSCs suppress T-cell proliferation via latent membrane protein 1 (LMP1)-mediated modification of glycolysis in nasopharyngeal carcinoma (NPC), an EBV-associated tumor^31^. We tried to confirm whether MDSC-like cells isolated from BM of EBV-infected mouse have a similar function, however, this cell population did not suppress T-cell proliferation. Expansion of MDSCs is thought to be tumor-dependent and this immunosuppressive cell type shows heterogeneity^32^. In addition, MDSCs likely require activation in order to exert their immune-suppressive function^33^. We infer that this activation signal was insufficient in our experimental setting. Besides, MDSC modulate not only T-cell suppression but also vasculogenesis, although we did not evaluate the latter function in the current study. Although we did not precisely define the functional role of this MDSC-like cell, our results clearly indicate that EBV infection has an impact on myelopoiesis.

Interestingly, our murine model recapitulates key aspects of the pathology observed in human patients. For example, increased GM-CSF and granulopoiesis in BM was confirmed in EBV-positive patients, but not in EBV-negative controls. In several clinical studies, an increased pre-treatment neutrophil-lymphocyte ratio (NLR) was associated with advanced disease and metastasis in several tumors, including NPC^34^. Although this index is not associated with patient survival, it might be a valuable prognostic marker in the case of EBV^+^ B-cell lymphoma. In summary, we proposed the model for EBV related diseases. EBV infection upregulates of GM-CSF and granulopoiesis which might be involved in immunosuppression leading to outbreak of the diseases. Further investigation of EBV-dependent alteration of myeloid development will further elucidate the interplay between this oncogenic virus and its host immune surveillance network.

## Materials and Methods

### Mice

NOD/Shi-*scid-*IL2rγ^*null*^ (NOG) mice were purchased from the Central Institute for Experimental Animals (Kanagawa, Japan) and were maintained under specific pathogen-free conditions at the animal facility of Tokai University. All animal experiments were approved by the Animal Care Committee at Tokai University (Kanagawa, Japan). The institutional guidelines for animal care and treatment in experimental investigations were used to perform the human care for the mice.

### Patients and specimens

This study was approved by the Institutional of Review Board of Tokai University, School of medicine. All human samples were analyzed accordingly. The information of human patient’s plasma were provided by Ken-Ichi Imadome. Bone marrow of human patient’s were provided by Kiyoshi Ando.

### Humanization of murine hematopoiesis

Hematopoietic humanization of NOG mice was conducted as described in a previous report^28^, and 4–5 × 10^4^ cells were injected into mice intravenously *via* the orbital vein 24 h post 2 Gy irradiation using an MBR-1505R X ray generator (Hitachi Medical, Tokyo, Japan). Approximately 3 months after the injection, the ratio of human-to-murine CD45 antigens was determined using a FACSVerse^TM^ multicolor flow cytometer (BD Biosciences, San Jose, CA) to evaluate the engraftment of human hemocytes.

### Generation of a murine model of EBV-positive lymphoproliferative disease (LPD)

Akata, a human lymphoma cell line that produces EBV, was treated with human IgG antibody in RPMI1640 medium (Nacalai Tesque, Kyoto, Japan) supplemented with 10% (v/v) fetal bovine serum (FBS), 50 U/ml penicillin and 50 mg/ml streptomycin in a flask (SUMILON MS-2105R; SANYO, Tokyo, Japan). The lytic phase was induced by treatment with anti-IgG antibody (Dako, Carpinteria, CA, USA) and virus was eluted into the supernatant. The supernatant was injected into mouse tails or orbital vein after filtration using a nylon filter net of 42μm pore size (SANSYO). Three to six weeks after the injection, dramatic weight loss occurred in mice (Fig. 1A). This is an index of LPD severity in this murine model. The mice were sacrificed before they succumbed to the disorder.

### Quantitative RT-PCR

Total RNA was extracted from hNOG mice using Sepasol-RNA I Super G (Nacalai, Kyoto, japan). Reverse transcription was performed using High Capacity cDNA Reverse Transcription Kit (Thermo Fisher Scientific). For q-PCR solution, THUNDERBIRD SYBR qPCR Mix (Toyobo, Osaka, Japan) was utilized. All reactions were performed in triplicate. q-PCR reaction was performed using Applied Biosystems^TM^ StepOne^TM^ Real-Time PCR System (Thermo Fisher Scientific). Used primers were as follows: h*GAPDH* forward (5′-CTGCACCACCAACTGCTTAG-3′), h*GAPDH* reverse (5′-TTCAGCTCAGGGATGACCTTG-3′); h*GM-CSF* forward (5′-CACTGCTGCTGAGATGAATGAAA-3′), h*GM-CSF* reverse (5′-GTCTGTAGGCAGG TCGGCTC-3′).

### ELISA

Murine or human blood samples were collected and transferred into a tube with heparin and centrifuged at 2,000 × g for 10 min. The supernatants were collected and stored at −20 °C to perform the ELISA. An ELISA kit (Meso Scale Japan, Tokyo, Japan) was utilized for the hGM-CSF analysis using thawed plasma. The absorbance was read using the MESO QuickPlex SQ 120 device (Meso Scale Japan). The detection range was 0.12–9,400.00 pg/ml, and the lower limit of detection (LLD) was 0.10 pg/ml. The calculated CV value was less than 10%.

### Flow cytometry

BM, spleen (SPL), and peripheral blood (PB) were obtained from mice. Single-cell suspensions were prepared from each tissue following standard procedures. Erythrocytes were hemolyzed using BD Pharm Lyse buffer (BD Biosciences). Leukocytes were stained with fluorochrome-conjugated human antibody/mouse antibody for 20 min at room temperature in FACS buffer. After washing the labeled cells in 1x PBS, the cells were re-suspended in FACS buffer. Fluorescent signals were detected with a FACSVerse^TM^ flow cytometer, and data were analyzed using FlowJo software (BD Biosciences). The following human antibodies were used to measure human engrafted hematopoietic cells and mouse hematopoietic cells: anti-human CD45-APC and anti-human CD66b-FITC were purchased from Beckman Coulter. Anti-mouse CD45-FITC, anti-human CD33-FITC, anti-human CD14-PE, anti-human CD11b-PE, anti-human CD41-PE, anti-human CD235a-FITC, anti-human CD3-FITC, anti-human CD19-APC were purchased from BioLegend.

### Morphological observation

The morphology of isolated human leukocytes was deduced from microscopic observation after May-Grünwald-Giemsa staining. Specimens were attached on a glass slide by centrifugation at 800 rpm for 5 min using a Cytospin instrument (Thermo Electric, PA, USA), and fixed in methanol that contained May-Grünwald solution (Merck, Darmstadt, Germany). May-Grünwald staining was performed for 3 min. Then, 10x PBS (pH 7.2) was added and incubated for 3 min. After washing the glass slides, Giemsa solution (Merck) diluted 10-fold in PBS was added to the specimen, then incubated for 15 min at room temperature. Finally, specimens were washed and dried, and microscopic observation was performed.

### Proliferation assay

Human CD4^+^ cells and CD8^+^ cells were sorted from peripheral blood using a flow cytometer. Sorted cells were co-incubated with EBV^+^ murine BM cells (hCD45^+^, hHLA-DR^−^, hCD33^+^) after stimulation with anti-CD3/28 beads (Dynabeads; ThermoFisher, USA). Various ratios (1:0, 8:1, 2:1, 1:1) of human T cells to murine BM cells were used. Unstimulated T cells were used as a control. Cell division was detected by reduced fluorescence intensity of CFSE (Dojindo Molecular Technology, Inc. Japan). Proliferation of hCD4^+^ cells and hCD8^+^ cells was then estimated.

### Statistical analysis

The data are indicated as mean value ± S.D. Student’s t test was performed to identify significant differences. A *p* value < 0.05 was used as the threshold for determining significant difference. *p < 0.05, **p < 0.01, ***p < 0.005 When the data indicate Lower limit of detection, the value for limit detection was used for the statistical analysis.

### Study approval

All animal experiments were approved by the Institutional Review Board of Tokai University. The animals received humane care as required by the institutional guidelines for animal care and treatment in experimental investigations. The Institutional Review Board of Tokai University, School of Medicine approved this study and all human samples were handled accordingly attesting to informed consent for study participation.

## Supplementary information

Supplemental information

Supplemental Figure and Table
