# Supplementary material for: Increased Granulopoiesis in the Bone Marrow following Epstein-Barr Virus Infection
Source: Sci Rep. 2019 Sep 17;9:13445. doi: 10.1038/s41598-019-49937-w (PMC6748920; doi:10.1038/s41598-019-49937-w)
Supplement: Supplementary file 1 — Supplemental information [file 41598_2019_49937_MOESM1_ESM.pdf]

Title

Increased Granulopoiesis in the Bone Marrow following Epstein-Barr Virus Infection

Authors

Yasuhiro Katahira[1], Hiroshi Higuchi[2], Hiromichi Matsushita[3], Takashi Yahata[4], Yuichiro Yamamoto[1], Ryo Koike[5], Kiyoshi Ando[6], Katsuaki Sato[7,8], Ken-Ichi Imadome[9], Ai Kotani[1, 6]

A list of Supporting Information

We prepared 4 supplementary informations as supporting information described below.

Supplementary **Figure S1**. Multi-lineage hematopoietic analysis of bone marrow between EBV infected and uninfected mice.

Supplementary **Figure S2**. T-cell suppression effect was not indicated by MDSC-like population in bone marrow of EBV-infected mice.

Supplementary **Figure S3**. Observed myeloid cell line progenitors in EBV infected mice BM.

Supplementary **Table S1**. Representative data of counted cell ratio in hCD66b+ population. hCD66b+ cells in bone marrow were isolated and stained to identify the cell contents. Observed cells were categorized to each cell lineage by morphological differences. Each cell numbers are average of count number in each mouse.
