# Supplementary material for: Increased Granulopoiesis in the Bone Marrow following Epstein-Barr Virus Infection
Source: Sci Rep. 2019 Sep 17;9:13445. doi: 10.1038/s41598-019-49937-w (PMC6748920; doi:10.1038/s41598-019-49937-w)
Supplement: Supplementary file 2 — Supplemental Figure and Table [file 41598_2019_49937_MOESM2_ESM.pdf]

Title

Increased Granulopoiesis in the Bone Marrow following Epstein-Barr Virus Infection

Authors

Yasuhiro Katahira[1], Hiroshi Higuchi[2], Hiromichi Matsushita[3], Takashi Yahata[4],
Yuichiro Yamamoto[1], Ryo Koike[5], Kiyoshi Ando[6], Katsuaki Sato[7,8], Ken-Ichi Imadome[9], Ai Kotani[1, 6]

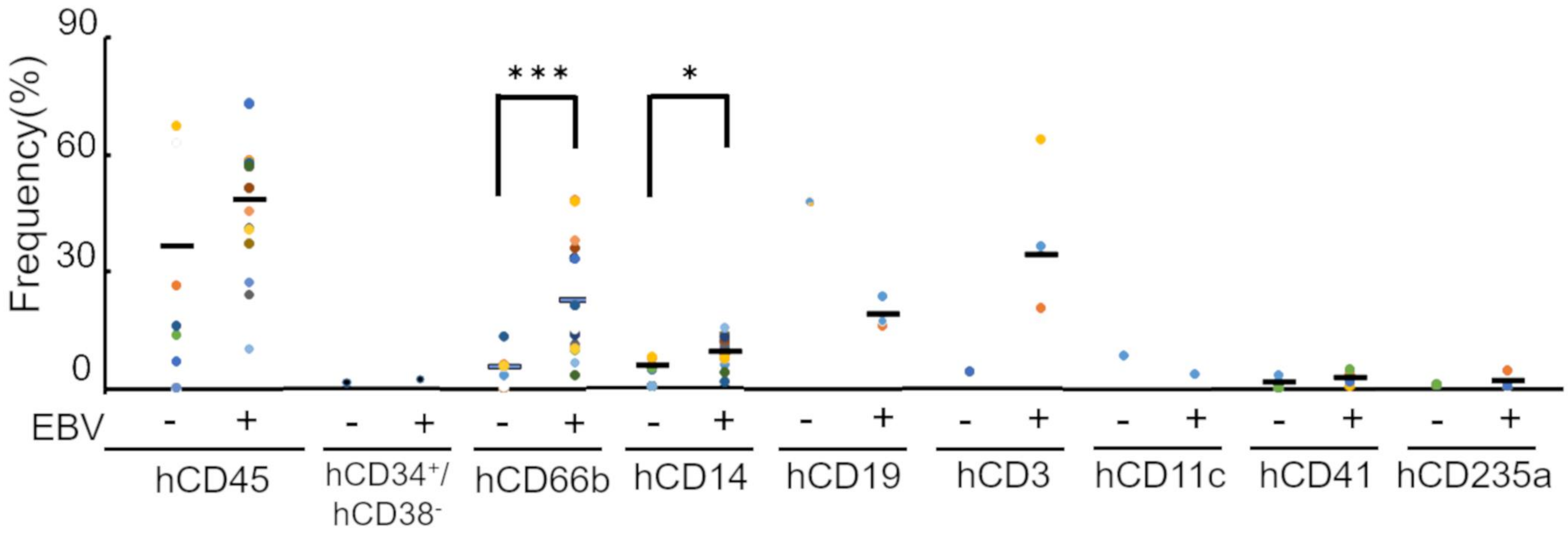

Figure S1: Multi-lineage hematopoietic analysis of bone marrow between EBV infected and uninfected mice. Each blood cells in bone marrow were detected and analyzed using flow cytometry. Described antibodies were used as marker for detection of each blood cells, respectively. hCD45=human leukocytes; hCD34^{+}/hCD38^{+}=primitive hematopoietic stem cells; hCD66b=granulocytes; hCD14=monocytes and Mφ; hCD19=B-cells; hCD3=T-cells; hCD11c=monocytes, Mφ, NK cells; hCD41=megakaryocytes; hCD235a=glycophorin A. *$p < 0.05$, **$p < 0.01$, ***$p < 0.005$.

Figure S2: T-cell suppression effect was not indicated by MDSC-like population in bone marrow of EBV-infected mice. (A) MDSC population were defined as hHLA-DR and hCD33^{+} population. MDSC-like population was detected from EBV-infected mice BM and SPL, but PB did not indicate it. (B) Purified MDSC-like population from BM of EBV-infected LPD mice was co-incubated with human T-cells. T-cells were stimulated by Dynabeads T-Activator CD3/CD28 (VERITAS, Japan). Stimulated T-cells (hCD4^{+} and hCD8^{+} population) division was not suppressed after incubation with MDSC-like population for 5 days.

(A)

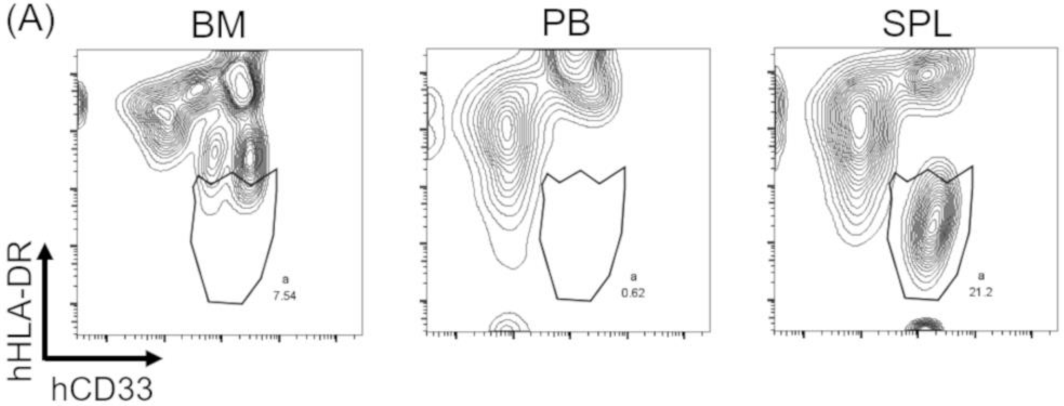

(B)

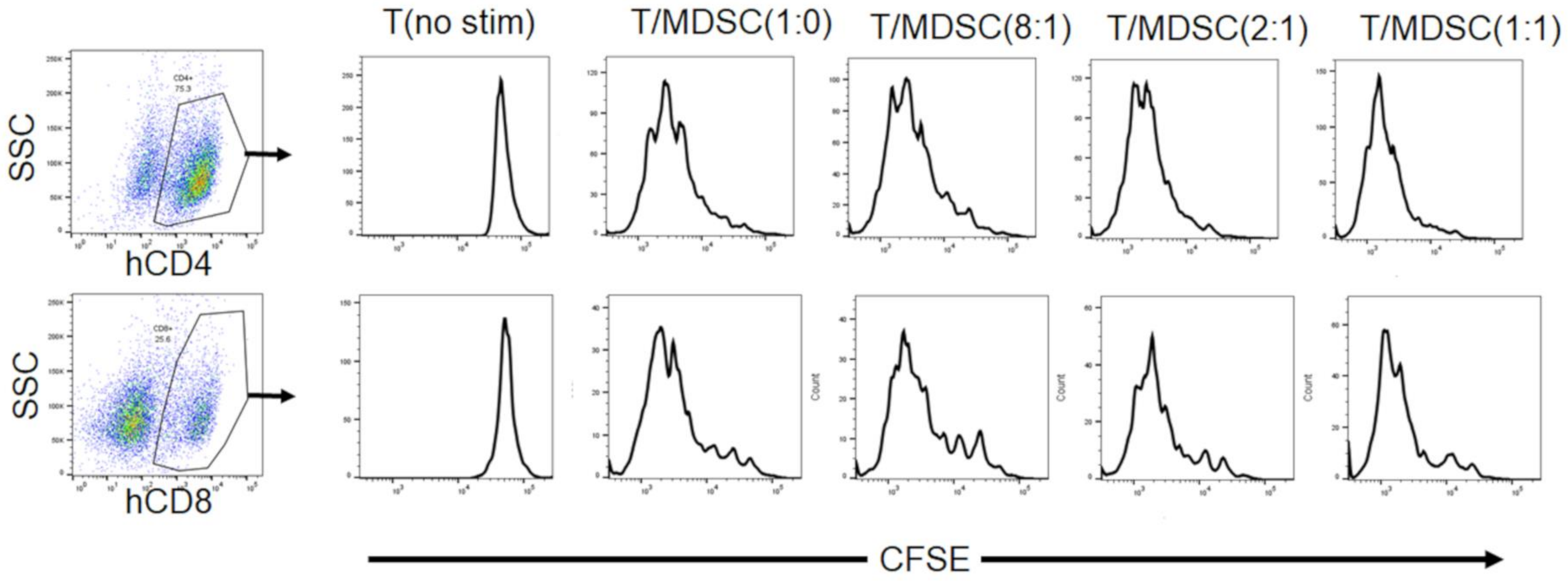

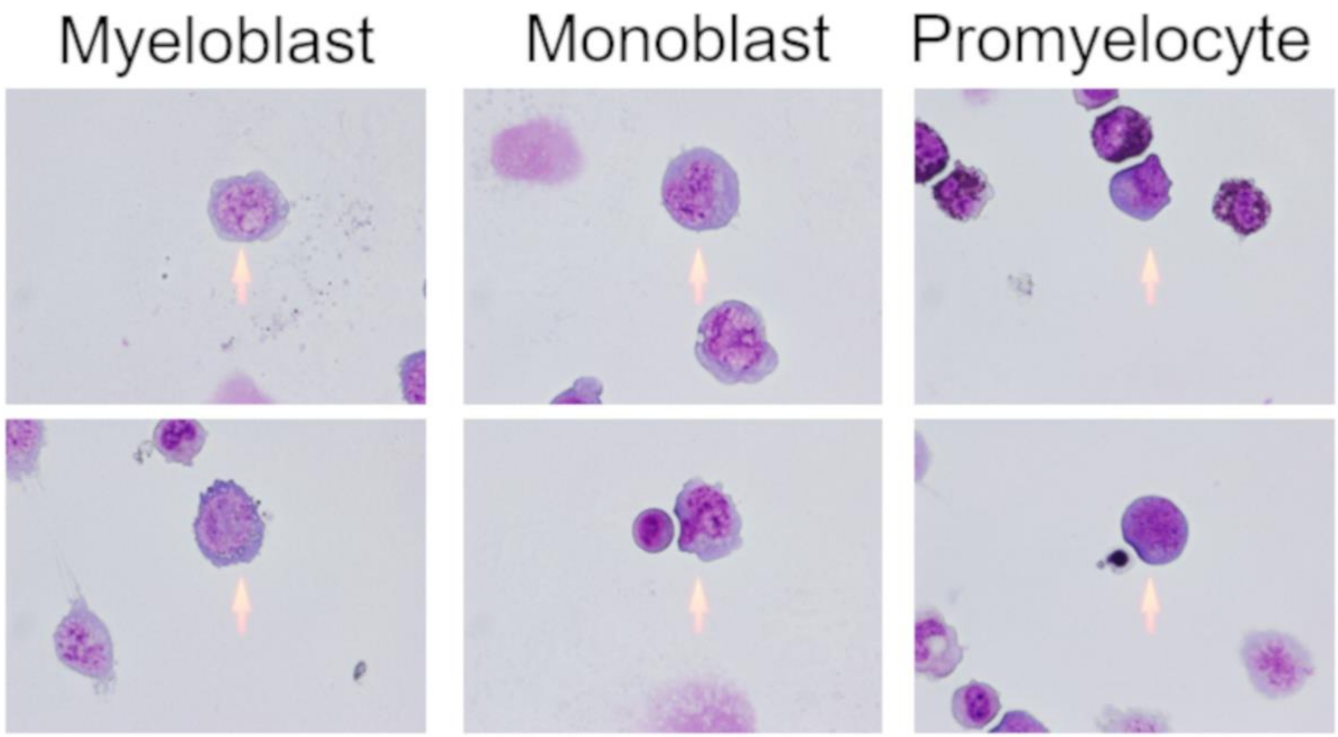

Figure S3: Observed myeloid cell line progenitors in EBV infected mice BM. Morphological features of cells were visualized by May-Grünwald Giemsa stain. Arrowhead shows representative each immature cells (Myeloblast, Monoblast and Promyelocyte).

Table S1. Representative data of counted cell ratio in hCD66b$^+$ population. hCD66b$^+$ cells in bone marrow were isolated and stained to identify the cell contents. Observed cells were categorized to each cell lineage by morphological differences. Each cell numbers are average of count number in each mouse.

	EBV	
Classificated cells	-	+
Myeloblasts	0.17	6.17
Promyelocytes	0.33	9.00
Myelocytes	5.83	22.00
Metamyelocytes	0.00	8.17
Band neutrophils	33.00	14.67
Segmented neutrophils	5.50	8.83
Monoblasts	0.00	6.33
Monocytes	8.83	9.83
Macrophages	0.00	0.33
Eosinophilic myelocytes	2.50	5.67
Eosinophilic metamyelocytes	4.17	1.83
Band eosinophils	2.00	1.67
Segmented eosinophils	0.83	0.83
Mastocytes	0.00	3.67
Lymphocytes	0.17	1.00
